# Androgen receptor signalling in Vascular Endothelial cells is dispensable for spermatogenesis and male fertility

**DOI:** 10.1186/1756-0500-5-16

**Published:** 2012-01-09

**Authors:** Laura O'Hara, Lee B Smith

**Affiliations:** 1 MRC Centre for Reproductive Health, University of Edinburgh, The Queen's Medical Research Institute, 47 Little France Crescent, Edinburgh EH16 4TJ, UK

## Abstract

**Background:**

Androgen signalling is essential both for male development and function of the male reproductive system in adulthood. Within the adult testis, Germ cells (GC) do not express androgen receptor (AR) suggesting androgen-mediated promotion of spermatogenesis must act via AR-expressing somatic cell-types. Several recent studies have exploited the Cre/lox system of conditional gene-targeting to ablate AR function from key somatic cell-types in order to establish the cell-specific role of AR in promotion of male fertility. In this study, we have used a similar approach to specifically ablate AR-signalling from Vascular Endothelial (VE) cells, with a view to defining the significance of androgen signalling within this cell-type on spermatogenesis.

**Findings:**

AR expression in VE cells of the testicular vasculature was confirmed using an antibody against AR. A Cre-inducible fluorescent reporter line was used to empirically establish the utility of a mouse line expressing Cre Recombinase driven by the Tie2-Promoter, for targeting VE cells. Immunofluorescent detection revealed expression of YFP (and therefore Cre Recombinase function) limited to VE cells and an interstitial population of cells, believed to be macrophages, that did not express AR. Mating of Tie2-Cre males to females carrying a floxed AR gene produced Vascular Endothelial Androgen Receptor Knockout (VEARKO) mice and littermate controls. Ablation of AR from all VE cells was confirmed; however, no significant differences in bodyweight or reproductive tissue weights could be detected in VEARKO animals and spermatogenesis and fertility was unaffected.

**Conclusions:**

We demonstrate the successful generation and empirical validation of a cell-specific knockout of AR from VE cells, and conclude that AR expression in VE cells is not essential for spermatogenesis or male fertility.

## Background

Androgens, produced by the testicular Leydig cells (LC), act as essential regulators of both the development of the male reproductive system and the initiation and maintenance of spermatogenesis in postnatal life (reviewed in [1,2]). Androgens bind to the androgen receptor (AR), a member of the nuclear hormone receptor transcription factor superfamily, to modulate gene transcription in target cells [3]. In the adult testis, Germ cells (GC), do not express AR, and GCs lacking a functional AR mature normally [4], consequently, androgens are believed to regulate spermatogenesis via androgen-AR signalling in AR-expressing testicular somatic cells. Recent exploitation of the Cre/lox system of conditional gene-targeting has elucidated the cell-specific role of AR in several key cell-types of the testis including Sertoli cells (SC) [5,6], LCs [7], GCs [8], Peritubular myoid cells (PTM) [9] and vascular smooth muscle cells (VSM) [10].

A further cell-type expressing AR and consequently responsive to androgen signalling are the vascular endothelial (VE) cells [11]. VE cells line the interior wall (*Tunica Intima*) of all vessels of the circulatory system. VE cells possess an anti-thrombotic surface which facilitates laminar blood flow, whilst permitting nutrients and macromolecules to flow out of the bloodstream through intercellular spaces, the result of endothelial cell contraction or active transport through the cells themselves by transcytosis. The endothelium also regulates angiogenesis, vasoregulation, and leucocyte trafficking into sites of injury during inflammatory responses (for review see [12]).

There remains some debate in the literature regarding the presence of AR within testicular VE cells, with studies arguing both for and against AR expression [13-15]. Given the wide-ranging impact of VE function on both the vascular system and peripheral organs, expression of AR within VE cells of the testicular blood vessels would raise the possibility that androgen-mediated modulation of endothelial cell function within the testis contributes to the regulation of spermatogenesis. To address this we first sought to unequivocally establish whether testicular VE cells express AR in vivo. We then exploited a conditional gene targeting approach to specifically ablate AR from endothelial cells of the vascular system to examine the impact of perturbed endothelial AR-signalling on spermatogenesis and male fertility.

## Findings

### Localisation of AR expression in the testicular vasculature

**Figure 1:**
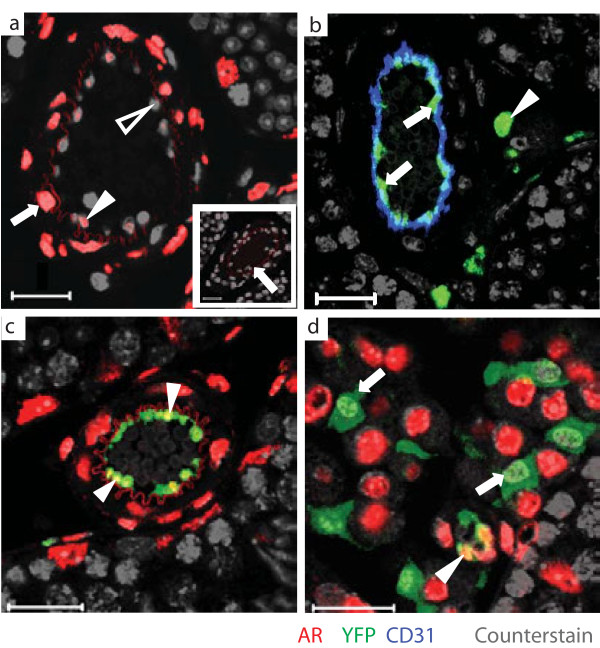
**A**: **Immunofluorescence detection localises AR to all smooth muscle cells of testicular blood vessels (arrow) and approximately 50% of vascular endothelial cells (solid arrowhead)**. AR is not detected in the remaining endothelial cells (outline arrowhead). Non-specific staining of arteriole elastic fibres occurs in even the absence of primary antibody (inset, arrow). **B**: Colocalisation of YFP immunofluorescence with the specific endothelial marker CD31 demonstrates that Tie2-Cre is active in endothelial cells of the testicular blood vessels (arrows). Tie2-Cre is also active in a proportion of testicular interstitial cells (arrowhead) **C**: Double immunofluorescence staining for AR and YFP demonstrates colocalisation of AR and YFP in a proportion of testicular vascular endothelial cells (arrowheads). **D**: YFP-expressing interstitial cells do not express AR (arrows). Scale bars are 20 μm.

To confirm that vascular endothelial (VE) cells of the testicular vasculature express AR, immunofluorescent detection of AR was performed on sections of testis taken from control adult animals (n = 3). Nuclear AR expression was confirmed within all smooth muscle cells of the vasculature as previously described [10]. In addition, AR localised to the nucleus of approximately fifty percent of testicular VE cells (Figure 1a).

### Empirical validation of a VE-specific Cre Recombinase mouse line

In order to specifically target AR in VE cells, a mouse line carrying a Cre Recombinase transgene driven by the promoter of the endothelial-specific angiopoietin receptor Tie2 was exploited [16]. To empirically establish the specificity of Cre Recombinase expression, (an essential requirement prior to any conditional gene-targeting experiment [17]), heterozygous Tie2-Cre males were mated to homozygous R26EYFPR females [18] and testes from offspring examined at adulthood. Expression of YFP was detected only in offspring inheriting the Tie2-Cre transgene (n = 3 per genotype). Within the testis of these animals, YFP expression was detected in VE cells of the blood vessel *Tunica Intima *(Figure 1b); however, a further population of interstitial cells also expressed YFP. Previous studies suggest these interstitial cells are likely to be macrophages which arise along with endothelial cells from a common Tie2-expressing progenitor population [19,20]. Double immunofluorescent analysis on adult testis sections (n = 3) using antibodies directed against AR and YFP clearly demonstrated that a proportion of the YFP-positive VE cells were also AR-positive (Figure 1c) but that YFP-positive interstitial cells do not express AR (Figure 1d); we therefore concluded that, although we would be deleting AR in both populations, the Tie2-Cre line had utility for specific ablation of AR-*function *in the Vascular Endothelium. Furthermore, no significant difference in bodyweight or seminal vesicle weight could be detected and spermatogenesis was confirmed to be normal in Tie2-Cre:R26YFPR animals (data not shown), demonstrating that the presence of the Cre Recombinase transgene did not itself induce a phenotype.

### Generation of VEARKO mice

**Figure 2:**
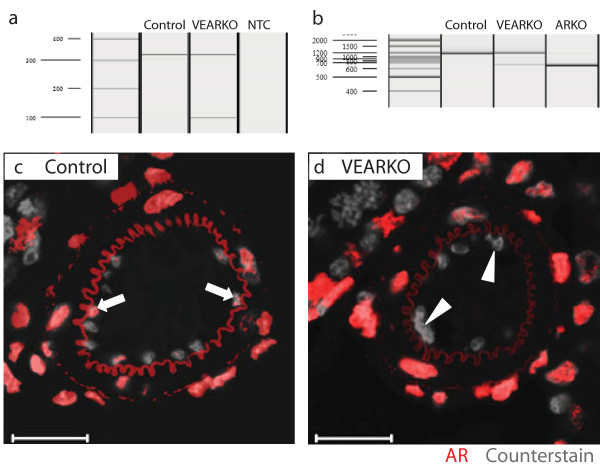
**A**: **VEARKO mice can be separated from controls through PCR interrogation of inheritance of Cre Recombinase**. 324 bp amplicon = internal positive control. 102 bp amplicon = Cre Recombinase. NTC = no-template control. B: PCR amplification of AR using primers spanning the floxed exon 2 on genomic DNA isolated from VEARKO and control testes demonstrates that a proportion of cells in the testes of VEARKO mice contain a recombined AR (612 bp). No recombination is observed in testes from control animals. Testes from total-ARKO mice, which demonstrate 100% recombination of AR, are included for comparison. C: AR expression is observed in endothelial cells of testicular blood vessels from Control mice (arrows); D: Conversely, no endothelial cells in testicular blood vessels of VEARKO mice express AR (arrowheads). Scale bars are 20 μm.

To generate Vascular Endothelial Androgen Receptor Knock-Out (VEARKO) mice and littermate controls, heterozygous Tie2-Cre males were mated to homozygous ARflox [5] females. Offspring were genotyped by PCR to establish inheritance of the Tie2-Cre transgene (Figure 2a).

PCR interrogation of genomic DNA extracted from testes of VEARKO mice confirmed ablation of AR from a subset of testicular cells (Figure 2b). No ablation was detected in littermate control testes (n = 5 per genotype). Immunofluorescent detection of AR protein on testis sections from VEARKO animals confirmed that ablation of normal AR-signalling within the testis was limited to VE cells, with AR ablated from all VE cells examined (n = 3) (Figure 2d). AR expression was retained in control littermates (n = 3).

### Phenotypic analysis of VEARKO mice

Comparison of bodyweight, seminal vesicle (SV) weight, and anogenital distance (a marker of androgen action during the masculinisation programming window [21]), revealed no significant difference between VEARKO males and controls (table 1) when examined in adulthood (postnatal day 57) (n = 3 per genotype); testes were fully descended into the scrotum.

**Table 1:** Adult VEARKO mice have normal body weight, AGD and SV weight

	Control	VEARKO
**Body weight**	24.2 ± 0.1 g	25.0 ± 1.0 g

**AGD**	15.6 ± 0.4 mm	15.5 ± 0.5 mm

**SV weight**	0.21 ± 0.0047 g	0.21 ± 0.0067 g

**Figure 3:**
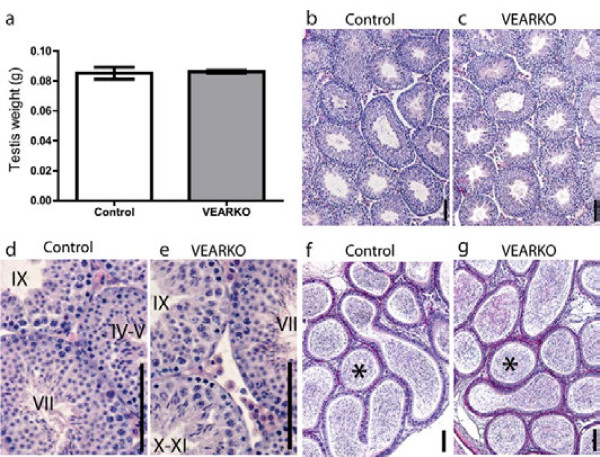
**A**: **Testis weight does not differ between control and VEARKO mice**. B-E: Spermatogenesis is normal in both Control and VEARKO testes, with all spermatogenic stages identified. F, G: morphologically mature spermatozoa are present in the cauda epididymides of both control and VEARKO animals (asterisks). Scale bars are 100 μm.

Gross phenotypic analysis revealed no significant difference in testis weight between VEARKO mice and controls in adulthood (Figure 3a). Blind-scoring of testicular sections from VEARKO and control mice (n = 5 per genotype) identified no phenotypic differences in testicular architecture with all stages of spermatogenesis present (Figure 3b-e) and morphologically mature spermatozoa detected in the cauda epididymis (Figure 3f-g). To determine fertility status, seven VEARKO males and five littermate controls were each housed individually with a 129SV/C57BL/6 JF1 female for five days. No significant difference was observed either in mating success (production of pups), or in average litter size. All seven VEARKO males and four of five control males sired pups with an average litter size of 7.857 ± 0.3401 (n = 7) and 8.250 ± 0.4787 (n = 4) respectively.

## Discussion

The testicular vasculature is known to be an important regulator of both testis development [20,22] and function [10]. The vascular endothelium which lines every blood vessel of the circulatory system and controls nutrient, macromolecule and immune cell entry into peripheral tissues, acts as an important regulator of the testicular micro-environment, and is known to respond to sex steroid hormone stimulation (for reviews see [23-25]). We have demonstrated that VE cells of the testicular vascular system express AR in vivo and thus are responsive to androgen-stimulation. However, surprisingly, AR expression was limited to approximately fifty percent of VE cells. Whether this represents two different functional populations of VE cells, a 'snap-shot' of AR expression cycling in all VE cells, or a response by certain cells to a specific localised signal requires further investigation.

Empirical validation of Cre Recombinase expression in the Tie2-Cre line provides a good example of why such validation is necessary (reviewed in [17]). Whilst we predicted VE expression of Cre Recombinase, further expression in an interstitial population of cells was unexpected. Based upon previous characterisation of the Tie2-Cre line, these cells are likely to be macrophages [19,20] which are known to physically localise in close proximity to AR-expressing Leydig Cells. Consequently the Tie2-Cre line is likely to have further utility as a testicular macrophage-targeting Cre Recombinase, this requires further empirical validation. For this study, however, the absence of detectable AR expression in these interstitial cells confirmed that the Tie2-Cre line had utility for ablating AR expression specifically from VE cells.

A combination of genomic PCR and immunofluorescent localisation confirmed specific AR ablation from the VE cells of the testis in VEARKO mice. However, unlike our previous study targeting AR in the smooth muscle cells of the testicular vasculature [10], we observed no significant difference in testis weight, seminal vesicle weight (a surrogate marker of circulating testosterone concentration), or any impact on spermatogenesis or fertility in VEARKO mice. We cannot rule out a role for VE-AR expression in aspects of testicular function other than support of spermatogenesis, however completion of spermatogenesis also requires production of androgens by normally functioning Leydig cells [26], and thus were there to be an impact on steroidogenesis, completion of spermatogenesis allows us to infer that this is also likely to be relatively minor. This requires further investigation. Nevertheless, this focused study unequivocally demonstrates successful generation of a mouse model lacking AR-signalling in VE cells of the circulatory system and confirms that AR-signalling via VE cells is not an essential requirement for spermatogenesis and male fertility.

## Methods

### Production of VEARKO and YFP reporter mice

Mice in which the AR has been selectively ablated from VE cells were generated using Cre/loxP technology. Male congenic C57BL/6 J carrying a random insertion of Tie2-Cre [16] were mated to C57BL/6 J female mice homozygous for a floxed AR [5]. The +/Cre, ARflox/y male offspring from these matings were termed 'VEARKO', whereas the +/+, ARflox/y littermates were used as controls, termed 'control'. A Cre-inducible yellow fluorescent protein (YFP) reporter line used to document the expression of the Tie2-Cre transgene was generated by mating Tie-Cre males to female mice homozygous for a YFP transgene inserted into the Rosa26 locus [18]. Sex and genotype ratios were all identified at the expected Mendelian ratios. The study was approved by The University of Edinburgh Ethics Committee and conducted under licensed approval from the UK Home Office (PPL: 60/3544).

### Fertility testing

Eight week old VEARKO male mice (n = 7) and littermate controls (n = 5) were singly housed with 129SV/C57BL/6 J females for a period of five days. Females were culled by inhalation of carbon dioxide and subsequent cervical dislocation, and fetuses counted ten days later.

### Recovery of reproductive tissues

Male mice were culled at post-natal day (d) 57 by inhalation of carbon dioxide and subsequent cervical dislocation. Body weight and anogenital distance were measured and mice were examined for any gross abnormalities of the reproductive system. Testes and SV were removed and weighed. Tissues were fixed in Bouin's fixative (Clin-Tech, Guildford, UK) for 6 h. Bouin's-fixed tissues were processed and embedded in paraffin wax, and 5-μm sections were used for histological analysis as reported previously [9]. Sections of testis were stained with hematoxylin and eosin using standard protocols and examined for histological abnormalities.

### Immunohistochemistry

Single immunofluorescent staining for AR (Santa Cruz sc-816) was performed on VEARKO and littermate controls and double fluorescent staining for AR/YFP (Abcam ab38689) or YFP/CD31 (Abcam ab28364) was performed on YFP reporter mice. Sections were deparaffinized and rehydrated, and high-pressure antigen retrieval was performed in 0.01 M pH6 citrate buffer for 5 min. Endogenous peroxidase was blocked using 3% hydrogen peroxide in methanol and non-specific antibody binding sites were blocked with a mixture of 20% normal goat serum/80% tris-buffered saline/5% bovine serum albumin (NGS/TBS/BSA). Sections were incubated overnight at 4°C with the primary antibody diluted in NGS/TBS/BSA. Immunostaining was detected using the appropriate secondary antibody (Goat anti-rabbit peroxidase (Dako P0448), or Goat anti-mouse peroxidase (Dako P0447)) diluted 1:200 in NGS/TBS/BSA incubated for one hour at room temperature, then fluorescein Tyramide Signal Amplification system ('TSA™', Perkin Elmer) diluted 1:50 in its own buffer to manufacturers instructions for 10 min at room temperature. If a second primary antibody was used, the process was then repeated from the peroxidase block onwards with the appropriate conditions and detection for the second antibody and use of Cyanine - 5 TSA™ for a different fluorescent wavelength to distinguish between the two antibodies. Slides were counterstained with propidium iodide (Sigma, UK, #P4170) and mounted with PermaFluor mounting medium (Thermo Scientific, UK). Images were captured using a LSM 710 confocal microscope (Zeiss) with Zen software. A minimum of three mice of each genotype were used and sections from VEARKO and control littermates were processed in parallel on the same slide, on at least two separate occasions. Appropriate negative controls were included to ensure that any staining observed was specific.

### PCR genotyping of mice

Mice were genotyped for inheritance of Cre Recombinase as previously described [9]. PCR amplification products were resolved using a QiaXcel capillary system (Qiagen, UK). An amplicon of 324 bp acted as a positive internal control whilst an amplicon of 102 bp indicated inheritance of the Cre Recombinase transgene.

### Determination of genomic ablation of AR

DNA was isolated from a piece of frozen testis using a Mouse tail genomic DNA kit (Gen-Probe Life Sciences Ltd, UK) according to the manufacturer's instructions and subjected to PCR amplification using primers GCTGATCATAGGCCTCTCTC and TGCCCTGAAAGCAGTCCTCT and Biomix Red Taq polymerase PCR kit (Bioline, UK). PCR amplification products were resolved using a QiaXcel capillary system (Qiagen, UK). An amplicon of 1142 bp indicated presence of a floxed AR whilst an amplicon of 612 bp indicated recombination between *loxP *sites and deletion of AR exon 2.

### Statistical analysis

Statistical analyses of weights, AGD and fertility testing were carried out in GraphPad Prism (version 5; GraphPad Software Inc., San Diego, CA, USA) using a 2-tailed unpaired *t *test.

## Competing interests

The authors declare that they have no competing interests.

## Authors' contributions

LBS conceived the study; LO carried out the experiments; LO and LBS wrote the paper. All authors read and approved the final manuscript.
